# Investigation of Thin Silicone Films on Opaque Solid Surfaces Using Coherent Raman Scattering Imaging

**DOI:** 10.1177/00037028251339495

**Published:** 2025-05-21

**Authors:** Julian Naser, George Sarau, Jan Wrege, Silke Christiansen

**Affiliations:** 1 Siemens Healthineers AG, Siemensstrasse 1, 91301 Forchheim, Germany; 2 Institute of Photonic Technologies, 9171 Friedrich-Alexander-Universität Erlangen-Nürnberg, Konrad-Zuse-Strasse 3/5, 91052 Erlangen, Germany; 3 Institute for Nanotechnology and Correlative Microscopy eV (INAM), Äussere Nürnberger Str. 62, 91301 Forchheim, Germany; 4 Fraunhofer Institute for Ceramic Technologies and Systems (IKTS), Äussere Nürnberger Str. 62, 91301 Forchheim, Germany; 5 Max Planck Institute for the Science of Light, Staudtstrasse 2, 91058 Erlangen, Germany; 6 Physics Department, 9166 Freie Universität Berlin, Arnimallee 14, 14195 Berlin, Germany

**Keywords:** Coherent Raman scattering, CRS, epi-coherent anti-Stokes Raman microscopy, E-CARS, thin films, contamination monitoring, Raman, polysiloxane

## Abstract

The measurement of thin films with a thickness in the nanometer range is challenging because it requires extensive sample preparation, vacuum condition, long measurement times or using test inks that additionally contaminate the surface. The detection of those films is crucial for production processes that rely on a boundary layer to create a proper interface like adhesive bonding, coating, or lithography in various industries like automotive, solar, energy storage and semiconductor manufacturing. Consequently, there is a need for quick, reliable measurement techniques with high sensitivity to ensure the technical cleanliness of the opaque surface. In this paper the feasibility of epi-detection with coherent Raman scattering (CRS) Imaging is investigated on different substrate materials and demonstrated to be a method for fast scanning of large nontransparent surfaces including chemical fingerprinting of the substances atop. Therefore, various samples with low surface energy filmic contaminations from polysiloxanes are produced and investigated with CRS Imaging, a technique mostly applied to biological samples with the novel use demonstrated here for surface contamination monitoring in material sciences.

## Graphical abstract

**Graphical abstract:**
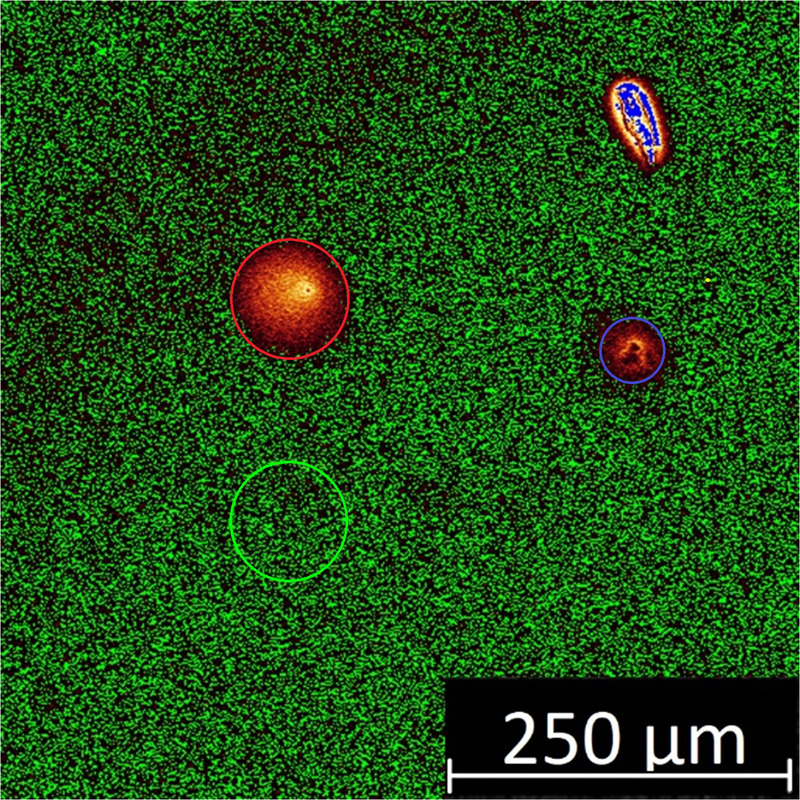
This is a visual representation of the abstract.

## Introduction

Surface contaminations have been studied for many decades in different industries to avoid their negative impact on product quality.^1^ For example, in the automotive manufacturing, where proper adhesion of the coatings is central to avoid defects in paintwork and corrosion, in the solar and energy storage manufacturing, where corrosion resistant coatings avoid a reduced lifespan, and the semiconductor manufacturing where, with electrical structures becoming smaller and smaller and the rising effect of contamination on the semiconductor yield, the relevance of the technical cleanliness steadily increases.^2–4^

To ensure technical cleanliness by detecting filmic contaminations non-destructively, a variety of techniques are available. The straightforward and cost-effective technique is contact angle measurement.^5^ Therefore, various test substances, regularly water and diiodomethane, are being applied to the contaminated surface, either by dropping them or using a pendant drop, and the contact angles of the developing drop shapes are measured. Subsequently various models are used to calculate the surface energy that is altered by the silicone contamination.^6,7^ However, this method has significant disadvantages such as the need to remove the test substances from the surface afterward, sensitivity to surface roughness and heterogeneity, which can both affect the accuracy of the measurements, and lack of information about the chemical nature of the contamination.

Other measurement techniques without altering the surface are shortly discussed in the following. First, Fourier transform infrared spectroscopy (FT-IR) offers a high chemical sensitivity but at a moderate spatial resolution.^8^ Second and third, X-ray photoelectron spectroscopy (XPS) and scanning auger microscopy (SAM) provide high surface sensitivity, non-destructive measurements with respect to chemical composition with high spatial and spectral resolutions, nevertheless with the need for sample preparation and high-vacuum conditions as the downside.^2,7,9^ Fourth, secondary ion mass spectrometry (SIMS) also provides high surface chemical sensitivity and high resolutions, both spatially and spectrally. Furthermore, the capability to detect light elements down to lithium and even hydrogen under optimized analytical conditions is advantageous. The major disadvantages are altering of the sample, high vacuum conditions, and challenging data interpretation.^10^ All those contactless techniques, also share the disadvantage of the high duration for large-area measurements, needed for nonuniform surface contamination, including sample preparation.

To accelerate the contamination detection the novel method coherent Raman scattering microscopy (CRSM) that originates from bio science was evaluated for its suitability in that specific application.^11^ The CRSM measurements can be performed with stimulated Raman scattering (SRS) or coherent anti-Stokes Raman scattering (CARS). The mechanisms of SRS and CARS have been investigated by Wang et al. and Djaker et al. and used to subtype Glioblastomas by Bae et al. to live-image hepatocytes by Rodriguez et al. and to distinguish normal, inflammatory, and colon carcinomas by Krafft et al.^12–15^ In this paper CARS is being used inter alia due to the technical limitations of our system that is equipped with CARS detectors only in the epi (reflection) direction that is needed for the opaque samples studied here. The laser modulation–demodulation scheme required to measure the energy transfer between the pump and Stokes beams, that SRS is based on, is not necessary for CARS detection, thus demanding simpler instrumentation as compared to SRS.^16^

**Figure 1.**
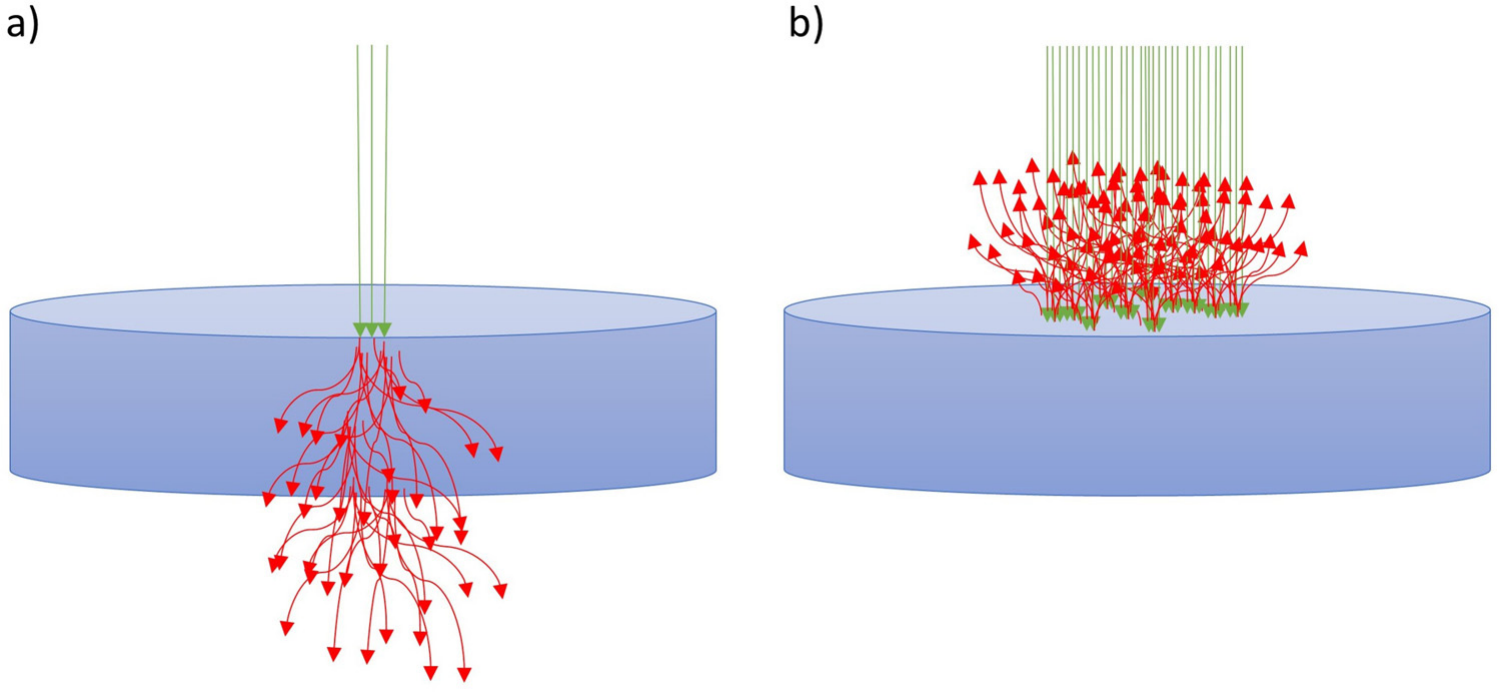
Path of photons for laser beam (green) and Raman photons (red) for (a) forward-CARS and (b) epi-CARS.^23^

In Figure 1, the path of photons is depicted for the two measurement geometries forward-CARS and epi-CARS. In the forward-CARS setting the photons penetrate the sample and are being detected after the transmission. By that the non-resonant background can overshadow the forward-CARS signal. This beam path is not applicable for samples that are not transparent at the specific laser wavelength, as those used in this study. Especially for those opaque samples the epi-CARS beam path is suited. There the photons are being reflected off the investigated surface and the detector is located above the sample. In literature, theoretical simulations of the beam path for forward and epi detection configurations have been conducted revealing that the distribution of the emitted Raman scattered photons maximizes along the incident laser beam for both measurement geometries. However, the distribution of the Raman photons around the optical axis differs, the photon distribution in the epi measurement setting is more focused on the center after the reflection, while the transmitted Raman photons are scattered more broadly after passing through the sample due to enhanced photon interaction with the sample internal structure.^17–22^

**Figure 2.**
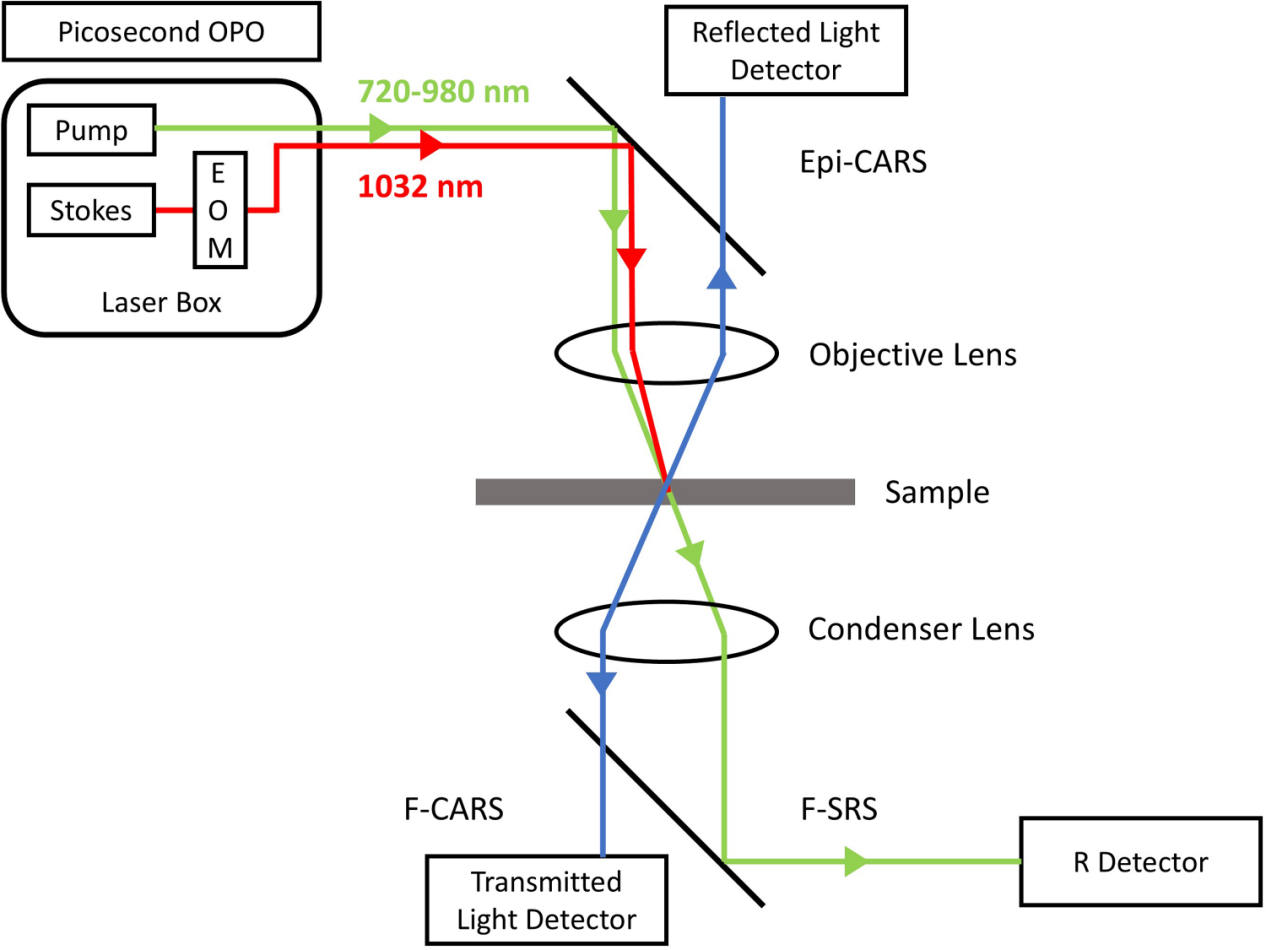
Schematic of the coherent Raman scattering microscopy beam path with different geometries and detectors.^28^

A detailed scheme of CRSM is shown in Figure 2, extended by the second beam path, the objective, and the condenser lenses. The two temporal and spatial overlapping short-pulsed laser beams lead to an amplification of the Raman signal. The blueshifted CARS signal has no fluorescence, but a non-resonant background is included, SRS has no non-resonant background. That difference improves the signal quality and opens new opportunities for measurements. To assess the signal quality, the signal-to-noise ratio (S/N) is used as a metric. The S/N ratio is a key factor to determine the quality and reliability of measurements. A high S/N is especially critical at investigations resulting in weak Raman signals against the background noise, e.g., thin films of low concentration. The aim is to obtain accurate Raman peaks that ensure reliable and reproducible chemical fingerprinting and quantitative analysis. Regularly the power of the signal is divided by the power of the noise, especially for image quality the ratio of signal mean S_Mean_ to the noise standard deviation σ_Noise_ is used. This ratio describes how much stronger the signal power is in comparison to the pixel-to-pixel variations and is used for the calculations in this paper.^24–27^

Research from Wang et al. indicates the theoretical feasibility of the detection of thin films down to one atomic layer on water in transmission with CARS.^29^ Zhang et al. published about single-molecule detection in reflection on specific, lithographically produced plasmonic substrates that enhance the CARS signal.^30^ Further research from Naser et al. investigated the detection of silicon thin films on transparent samples with Coherent Raman Scattering Imaging in the forward geometry, thus measuring in transmission.^31^ If measurement in transmission is not possible due to the opaque nature of the samples, extensive sample preparation involving thinning the specimens down to 10 to 100  µm is applied, until they are transmissive.^32^ Especially for sensitive samples this process must be gentle and is subsequently time consuming, to avoid altering of the investigated area.

**Figure 3.**
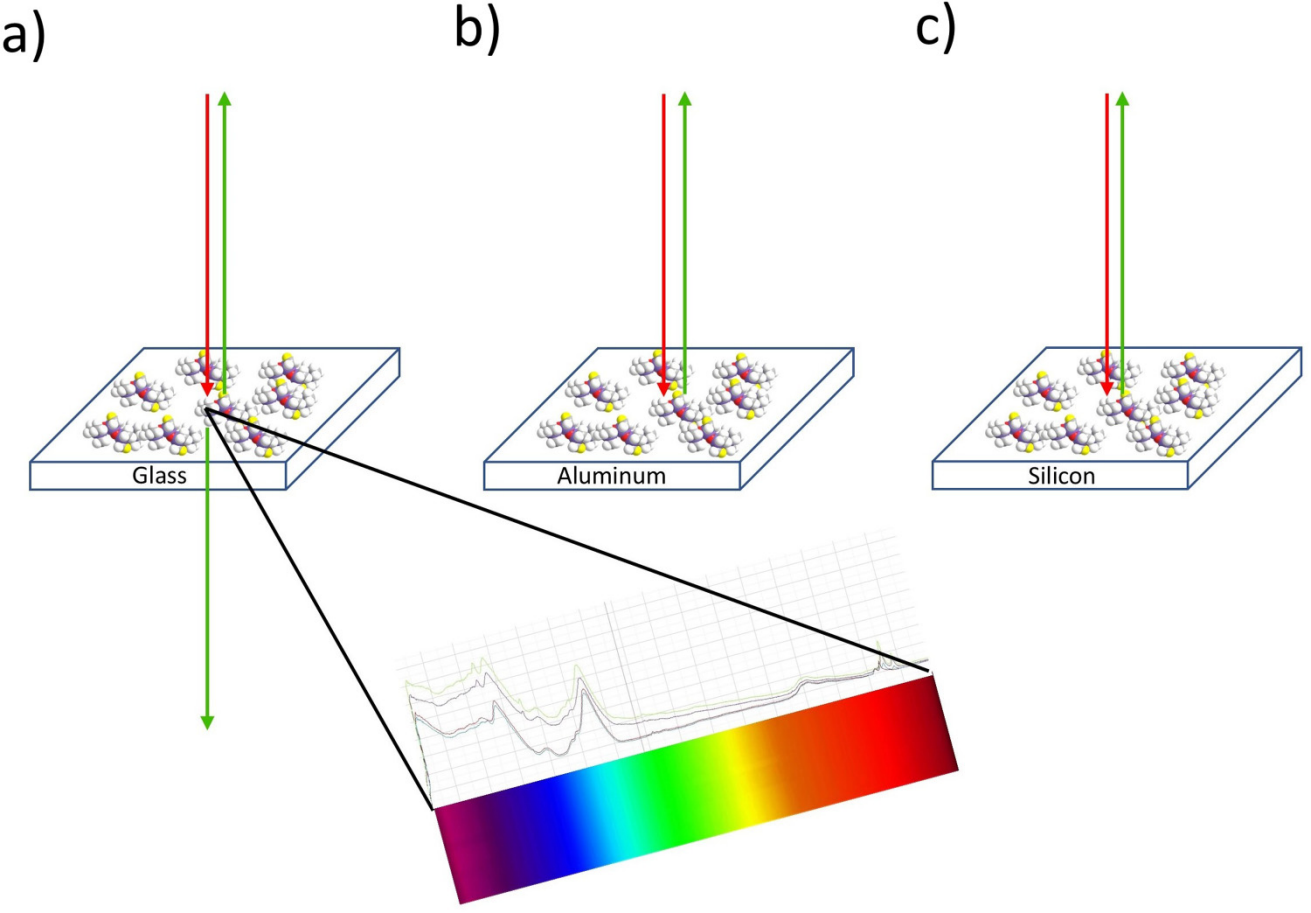
Overview of the measurement geometry for (a) glass sample with forward- and epi-CARS, (b) aluminum sample with epi-CARS, and (c) silicon sample with epi-CARS.

To avoid labor-intensive work and the use of specific, lithographically produced plasmonic substrates for low-concentration detection, this article demonstrates measurements in reflection on opaque samples without sample preparation, by establishing a method using the epi-detection geometry. This method is especially relevant for the practical use in production environments, where no sample preparation is possible, and the measurement time is crucial. To evaluate its capabilities, measurements are performed on different substrates including thin silicone films on the surface of aluminum, glass, and silicon materials. Figure 3 displays these specific samples along with the used measurement geometry.

These substrates were selected to cover a variety of material properties as employed in different industries: (i) Glass as an amorphous isolating material with low reflectivity and suitable for transmission measurements (Figure 3a), (ii) silicon as a crystalline semiconducting material with widespread use in electronics and photonics for measurements in reflection (Figure 3b), and (iii) aluminum also for measurements in reflection as a conducting material with high reflectivity and a distinct non-resonant background (Figure 3c). This broad range of material properties can provide valuable insights into different aspects of the light–material interaction behavior in forward and epi-CARS for the detection of filmic contaminations.

## Experimental

### Sample Preparation

Three types of samples are investigated. As transparent samples, standardized glass microscopy slides according to ISO 8037/1 are utilized. The opaque samples are aluminum tension rods made from AlMg_3_. The third type of samples are silicon wafers, which are opaque at the used laser wavelength, coated with 100  nm silicon dioxide (SiO_2_). The coating is transparent at the used wavelength for the measurement, so it is a combination of transparent in the first 100  nm and opaque in the following.

The different samples are prepared by pipetting 10 µL solution with 0.001 percent to 10% polysiloxane diluted in *n*-Heptane >99% on the surface. The drop spreads until it covers an area of 1 cm² and evaporates, leaving the thin PDMS films. With the density of 0.97 g⋅cm^–^³ the average film thickness can be estimated. Table I shows the calculated average values for the film thickness and surface coverage linked to the concentration of the solution pipetted on the surface of the sample. In practice the film thickness varies, especially because of the coffee ring effect, which leads to higher film thicknesses at the circumference of the droplet due to the capillary flow. The evaporating liquid at the perimeter is replaced by liquid from the center, carrying the solved PDMS with it.^33^ By using the tenfold difference between two successive concentrations and by measuring in the center those variations are negligible, and the calculated value can be used as an approximation.

**Table I.** Calculated film thickness and average surface coverage dependent from concentration.^31^

Concentration PDMS (%)	Average film thickness (nm)	Average surface coverage (μg⋅cm^−2^)	Detectability with spontaneous Raman
10	10 000	1 000	Yes
1	1 000	100	Yes
0.1	100	10	Yes
0.01	10	1	No
0.001	1	0.1	No

### Characterization Methods

The spontaneous Raman measurements took place on the Horiba LabRAM Explora Raman spectrometer with the Syncerity OE detector. The measurement time was 225 seconds (15  s with 15 accumulations), the objective was the Olympus 50X LWD, the grating was 300 lines/mm, the laser wavelength was 532  nm, and the laser power was 15.275  mW. The step size was 2.04 cm^–1^, and the spectrum was taken from 0 to 3200 cm^–1^.

The CRS measurements for the main investigation were performed on a Leica Stellaris SP8 CRS coherent Raman scattering microscope. The objective was the HC PL Fluotar 10×/0.30 N.A. With that an area of 1162.5 × 1162.5  µm was measured with a scan speed of 400 kHz, equaling a pixel dwell time of 3.1625 µs. The investigated area was 512 × 512 pixel resulting in a pixel size of 2.275  µm. The wavelength of the generated CARS signal λ_CARS_ can be calculated with Eq. 1 and spreads from 636.01 to 655.84  nm,^34^ with the exciting pump beam wavelength from 787  nm to 802  nm combined with the 1032  nm Stokes beam.

(1) $$\begin{array}{cc} & \lambda_{\mathrm{CARS}}=\frac{1}{\frac{2}{\lambda_{\mathrm{pump}}}-\frac{1}{\lambda_{\mathrm{stokes}}}}\\ \;\mathrm{Constraint}: & \;\;\;\;\;\;\;\lambda_{\mathrm{CARS}}<\lambda_{\mathrm{pump}}<\lambda_{\mathrm{stokes}} \end{array}\;\;\;\;\;\;$$

in which λ_CARS_ equals the wavelength of the generated CARS signal, λ_pump_ is the wavelength of the pump laser, and λ_stokes_ is the wavelength of the Stokes laser.

Equation 2 allows to calculate the wavenumbers of the excited vibrational band; those range from 3016.56 to 2778.91 cm^–1^.^34^

(2) $$\begin{array}{c} \;\;\;\;\;\;\;\;\;\;\;\;\;\;\;\;\;\;\;\;\;\;\;\;{\tilde{v}}_{\mathrm{vib}}=\frac{1}{\lambda_{\mathrm{pump}}}-\frac{1}{\lambda_{\mathrm{stokes}}}\\ \mathrm{Constraint}:\;\;\;\;\;\;\;\;\;\;\;\;\;\;\lambda_{\mathrm{pump}}<\lambda_{\mathrm{stokes}} \end{array}$$

in which ${\tilde{v}}_{\mathrm{vib}}$ is the wavenumber of the excited vibrational band, λ_pump_ is the wavelength of the pump laser, and λ_stokes_ is the wavelength of the Stokes laser.

### Theoretical Considerations

In earlier studies the detection limits of polysiloxanes with a viscosity of 5000 have been determined.^31^ A deciding factor for the detectability is the chain length of the polysiloxane molecule. Table II shows the viscosity, the chain length, and the molecular weight of the used polysiloxanes. The lower the chain length, the higher the volatility and the potential impact of polysiloxanes on the surface properties, which are important for industrial production processes like adhesive bonding, coating, and lithography.^35–38^

**Table II.** Viscosity, chain length, and molecular weight for various polysiloxanes.^39^

PDMS	Viscosity (mPas)	Chain length	Molecular weight (u)	Type of polymer according to molecular weight
M10	10	15	1.250	Low
M100	100	80	6.000	Middle
M500	500	300	22.500	Middle
M1000	1000	400	30.000	Middle
M5000	5000	650	48.000	Middle

To estimate the detection limits for polysiloxanes with different chain length, polysiloxanes with a viscosity of 10 mPas, 100 mPas, 500 mPas, 1000 mPas, and 5000 mPas at 25 °C were compared with respect to their signal strength using spontaneous Raman. First some theoretical considerations and background are given to understand qualitatively the variations in the Raman intensity, while the results are presented in the next section.

Equation 3 theoretically calculates the intensity of Raman scattered light^34^ of a sample. A higher chain length equals a higher number of scattering molecules (*N*) in the analyzed area in combination with an increased reduced mass of the vibrating atoms (µ). According to that, a higher chain length leads to a directly proportionally stronger Raman signal.

(3) $$\begin{array}{rl} & I(v{)}_{R}=\frac{2^{4}\pi^{3}}{45\cdot 3^{2}\cdot c^{4}}\cdot \frac{hI_{L}N{\left(v_{0}-v\right)}^{4}}{\mu \nu \left(1-e^{-\frac{hv}{kT}}\right)}\\ & \;\;\;\times [45{\left(\alpha_{a}^{{}^{\prime }}\right)}^{2}+7{\left(\gamma_{a}^{{}^{\prime }}\right)}^{2}] \end{array}$$

in which *c* is the speed of light, ℎ is Planck's constant, *I*_L_ is the excitation intensity, *N* is the number of scattering molecules, ν is the molecular vibrational frequency in Hertz, ν_0_ is the laser excitation frequency in Hertz, μ is the reduced mass of the vibrating atoms, *k* is the Boltzmann constant, *T* is absolute temperature, α'_a_ is the mean value invariant of the polarizability tensor, and γ'_a_ is the anisotropy invariant of the polarizability tensor.

Table III assigns the molecular vibrations at specific wavenumbers to their associated bond, mode, and symmetry. In this experiment two peaks have been closer investigated. The peak at 2906 cm^–1^, where the symmetric stretching of the carbon–hydrogen bond in the A1 and A2 symmetry is located, and the 2966 cm^–1^ peak that is mainly associated with the asymmetric stretching in the A2, B1, and B2 symmetry of the carbon–hydrogen bond and marginally with the symmetric stretching in the A1 symmetry.^41,42^

**Table III.** Assignment of wavenumbers to bond, mode, and symmetry for polydimethylsiloxane (PDMS).^42^

Raman (cm^–1^)	Bond	Mode	Symmetry
2966	C–H	Symmetric stretching	A1
2966	C–H	Asymmetric stretching	A2
2966	C–H	Asymmetric stretching	B1
2966	C–H	Asymmetric stretching	B2
2906	C–H	Symmetric stretching	A1
2906	C–H	Symmetric stretching	B2
1411	C–H	Asymmetric stretching	A1
1411	C–H	Asymmetric stretching	A2
1411	C–H	Asymmetric stretching	B1
1411	C–H	Asymmetric stretching	B2
1263	C–H	Symmetric stretching	A1
1263	C–H	Symmetric stretching	B2
861	CH3	Rocking	A1
788	C−Si−C	Asymmetric stretching	B2
680	CH3	Rocking	B2
489	Si–O	Symmetric stretching	A1
190	C−Si−C	Scissoring	A1
190	C−Si−C	Rocking	B1
190	C−Si−C	Wagging	B2
165	C−Si−C	Twisting	A2

## Results and Discussion

**Figure 4.**
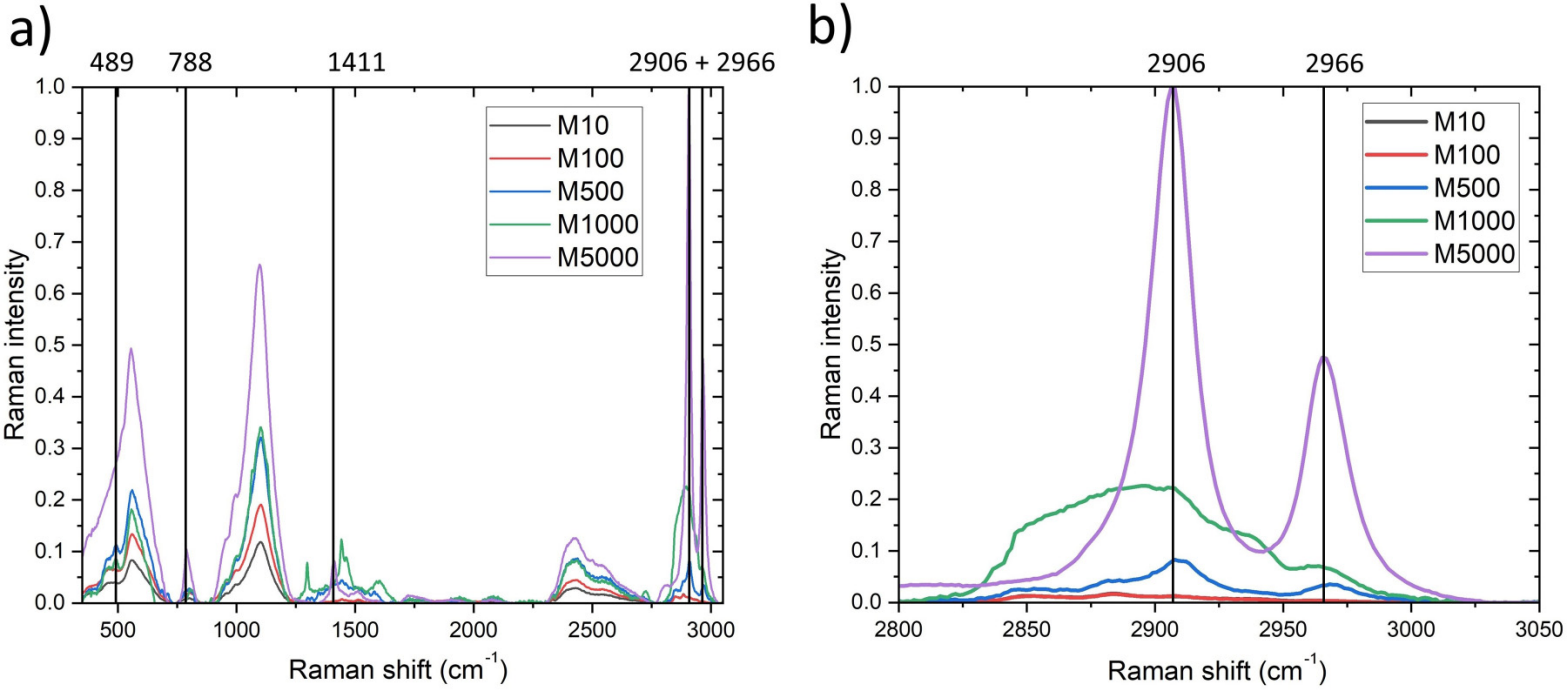
(a) Complete spontaneous Raman spectra for polysiloxanes with different chain length, and (b) excerpt of the 2800–3050 cm^–1^ spectral range with specific PDMS peaks for different chain length.

Figure 4a exhibits the complete spontaneous Raman spectra resulting from the preliminary PDMS chain length investigation described in Theoretical Considerations section, with the key peaks indicated by vertical lines. Table III assigns the peaks to the corresponding chemical bond, mode, and symmetry. At 489 cm^–1^, the symmetric stretching of the Si–O bond results in detectable peaks for M1000 and M500. The spectra of M100 and M10 overlap. At 788 cm^–1^, the asymmetric stretching of the C–Si–C bond results in a distinct peak that is visible for the M5000 viscosity. For the lower viscosities, the peaks are weak. At 1411 cm^–1^ the asymmetric stretching in the A1, A2, and B1 symmetry of the C–H bond results in a distinct peak for M5000, while the lower viscosities are not detectable.

Figure 4b focuses on the spectral region 2800–3050 cm^–1^ where the most pronounced peaks, resulting from the C–H bond, are located. The spectra of M10 and M100 overlap. Weak peaks are detectable with spontaneous Raman at 2906 cm^–1^ and 2966 cm^–1^ down to the M500 viscosity. At M5000 the two peaks are clearly pronounced with a high intensity. To sum it up the expected results resulting from Eq. 3 can be replicated, the peaks of the polysiloxanes with a higher viscosity are more distinct, clearer visible, and easier to detect.^40^ The polysiloxane with the M5000 chain length has been selected for the following investigations to determine the S/N at various surface coverages, measurement geometries, and substrate materials.

**Figure 5.**
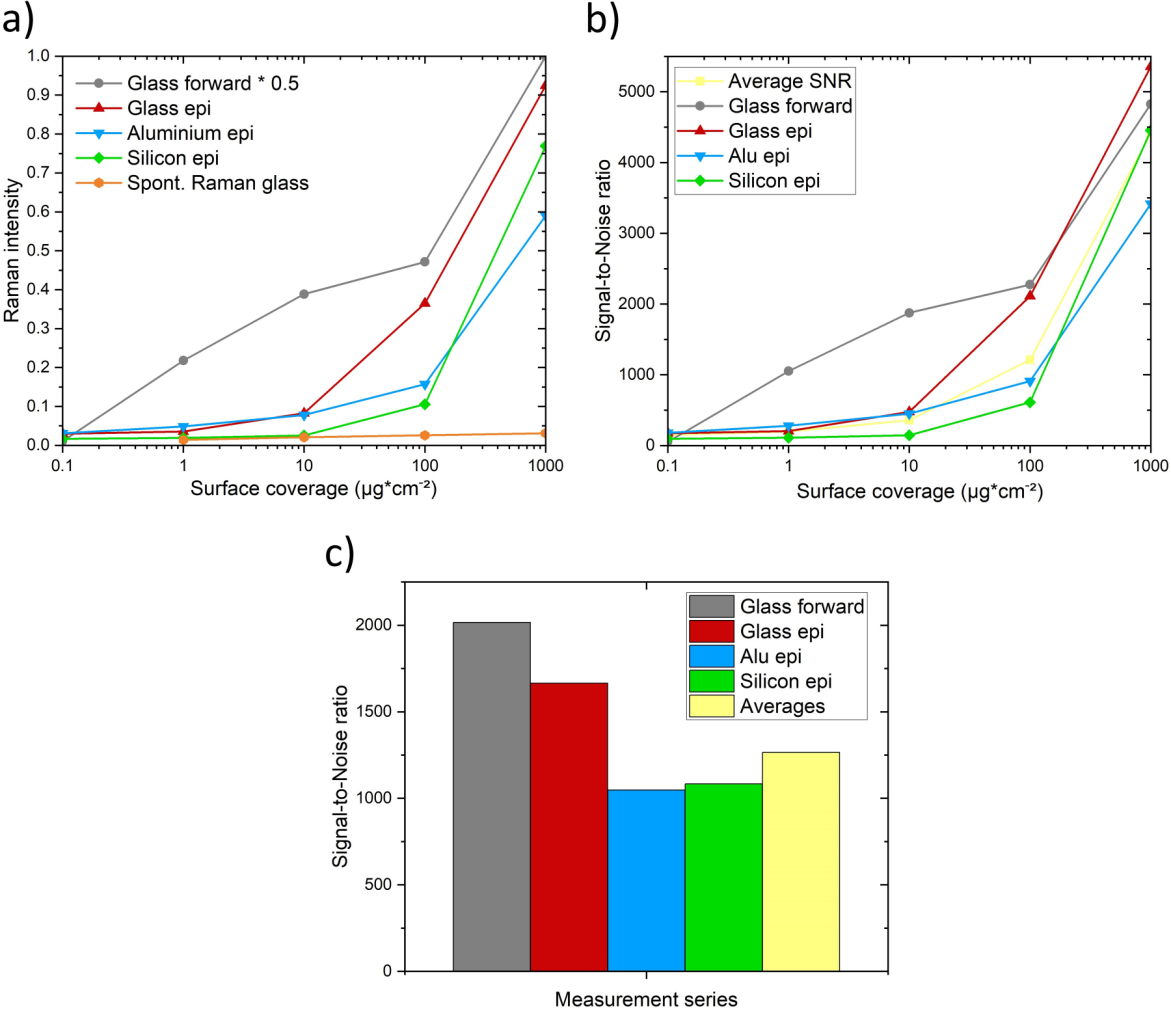
(a) Comparison of CARS signal strength for glass forward, glass epi, aluminum epi, silicon epi and spontaneous Raman on glass, normalized for laser power, detector sensitivity, detector gain, and baseline corrected. (b) CARS S/Ns of different surface coverages and materials and (c) CARS S/Ns of different measurement settings.

The S/N is crucial in evaluating the quality and reliability of measurements. The main factors influencing the S/Ns in our study are discussed in the following. First, the measurement geometry, with forward measurements offering higher S/Ns as compared to epi–measurements showing lower S/Ns. The reasons for higher S/Ns in the forward setting are a lower optical loss, a higher number of Raman scattered photons because of the larger interaction volume of the material being excited, and less Rayleigh scattering. Second, the reflectivity of the substrate material in forward measurements should be ideally low to ensure most of the photons can interact with the sample and can be detected after penetrating the sample. For epi measurements in reflectance also a low reflectivity is advantageous because it reduces the amount of stray light. Glass has the lowest reflectivity of the three materials with less than 10% of incident light being reflected at most visible wavelengths.^43^ It is followed by silicon with 30 to 40%.^44^ Aluminum has the highest reflectivity with more than 90%.^45^ Third, the non–resonant background decreases the S/N. It is least pronounced for glass.^46^ For silicon it is moderately distinct.^47^ And for aluminum it is most evident because of the high electronic polarizability.^48^ The results of the Raman and S/N measurements that are presented in Figure 5 are matching with those considerations.

Figure 5a summarizes the Raman measurements of PDMS M5000 at 2966 cm^–1^ function of its concentrations on different substrates. It compares the spontaneous Raman intensity (orange) with the CARS intensity in the epi-direction on glass (red), aluminum (blue), and silicon (green), and a glass sample (gray) measured in the forward direction, which signal intensity is divided by two to better illustrate the graph. While in the CARS measurements all concentrations were detectable, with the spontaneous Raman the lowest concentration was not observable. The advantage of the forward measurement geometry is especially visible at the surface coverage of 1 and 10 µg⋅cm², but the signal intensity is superior at all measurement points. Otherwise, the epi-detection geometry performed with regard to the detectability similar to the forward-detection geometry. Other publications that compared forward and epi measurements reported intensity differences that are 6 to 47 times higher, although in those studies, thinner substrates than our 1 mm-thick glass slides were used, that did not attenuate the signal to that great extent.^49–53^

Figure 5b shows the average S/Ns of the different surface coverages for the measurement series. The overall trend is similar to that in Figure 4a. At the 1000 µg⋅cm^–2^ coverage the average S/N is 4408, and it varies between 3416 for aluminum epi and 5355 for glass epi. The 100 µg⋅cm^–2^ average S/N is 1212 and is spreading between 611 for silicon epi and 2275 for glass forward. 10 µg⋅cm^–2^ average S/N is 358, with values between 147 for silicon epi and 1875 for glass forward. For 1 µg⋅cm^–2^ the average S/N is 198, the highest value is 1051 for glass forward and the lowest value is 110 for silicon epi. At the lowest coverage with 0.1 μg⋅cm^−2^ the average S/N is 149, differing between 178 for aluminum epi and 52 for the glass forward measurements. The S/N of 52 is the lowest value, which shows that the measured signal remarkably outweighs the noise standard deviation. Thus, the higher the coverage, the higher the S/N resulting from the over proportional higher signal intensity with the noise standard deviation staying low.

Figure 5c presents the S/N averages over all coverages assigned to the measurement settings. The transmission geometry at the glass sample offers the highest S/N with a value of 2015. The comparison of the materials shows that in the epi geometry also glass offers the highest S/N with 1664, followed by silicon with 1083 and aluminum with 1047. The average S/N of 1265 confirms that the measurement results of all tested arrangements are reliable and not remarkably altered by noise. The measurement time of the CARS measurement is remarkably reduced from 225 seconds per spectrum per pixel to 140 seconds for the spectra of 262144 pixels. That reduction of measurement time enables large-area scans as shown in the next paragraph.

**Figure 6.**
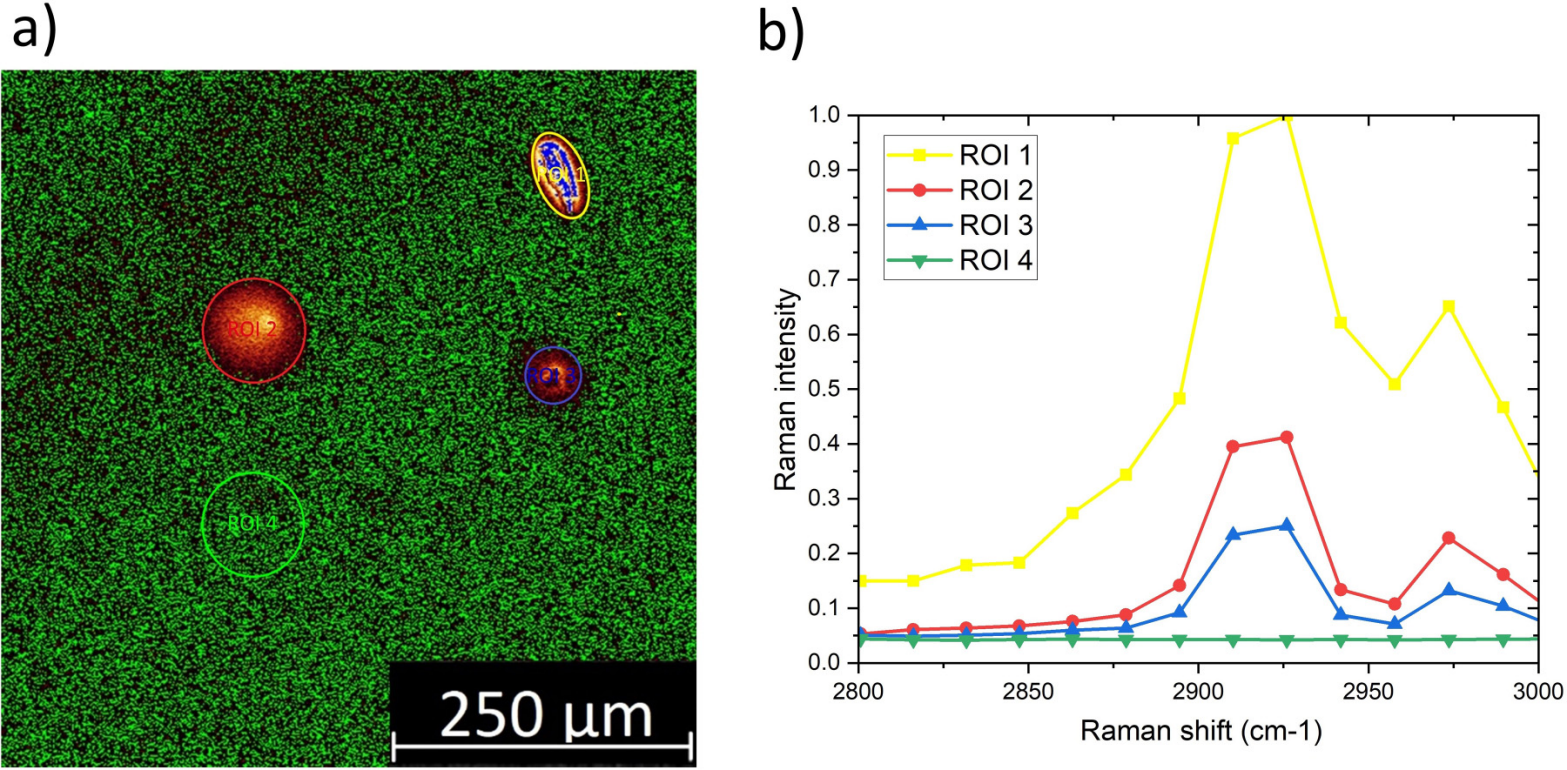
(a) Epi-CARS imaging of PDMS contaminated silicon surface. (b) CARS spectra for clean and various contaminated areas with the specific peaks.

Figure 6a presents epi-CARS imaging of a PDMS-contaminated silicon surface, where defined PDMS spots are visible. The region of interest (ROI) 4 is the signal of the uncontaminated surface. ROIs 1–3 are PDMS droplets with different thicknesses. The corresponding spectra in Figure 6b exhibit a direct correlation between the layer thickness and the corresponding signal intensity at the peaks around 2966 cm^–1^ and 2906 cm^–1^. In future studies this correlation and the thickness measurement capabilities will be investigated closer. The aim is to offer the opportunity to be able to straightforwardly, without any sample preparation, vacuum condition or altering of the sample, determine the spatially resolved thickness of thin films to assess if the contamination is in the range that is critical for the subsequent production stages. The spatial resolution is the pixel size of 2.275  µm and the height resolution has to be determined and is expected to be in the nm range with an increase in the objective’s magnification.

This shows that although the substrate is in focus, the higher the thickness of the contamination, the larger the Raman intensity that is detectable because the used 10x objective probes a large optical thickness. Furthermore, the spectra show the fingerprinting capabilities to identify unknown substances by taking a spectrum and assigning the peaks by hand based on a literature review or by using commercially available software including the respective Raman spectral reference databases. An example is the KnowItAll Analytical Edition Software with the KnowItAll Raman Spectral Library from Wiley Science Solutions.

## Conclusion

In this paper the feasibility of utilizing the coherent anti-Stokes Raman scattering technique in coherent Raman scattering microscopy to detect thin films of organic contaminants on opaque and transparent samples has been presented. Therefore, the epi-geometry has been used, where the backscattered Raman photons are counted. It is a strong and nondestructive technique for samples that are not suited for forward measurements due to their thickness and lack of transparency. By that the labor-intensive thinning of samples down to 10 to 100  µm to make them transmissive can be avoided. In a preliminary investigation the influence of the chain length of the polysiloxanes on its Raman signal has been investigated and the link that a higher chain length leads to higher peaks and easier detectability has been shown.

Afterwards, substrates of different materials with different thin films were prepared, equaling to an average coverage of 0.1–100 µg⋅cm^–^² PDMS, to determine the detection limits of the coherent Raman technique. A closer investigation of the results and the calculated S/Ns showed that the signal quality of the forward CARS measurements is superior, but the epi-CARS S/N is sufficient to obtain reliable results. In comparison to spontaneous Raman the CARS measurement time is reduced by orders of magnitudes. By that large-area scans become possible within a reasonable timeframe. One cm² can be scanned in minutes instead of days, thereby also In-Line production monitoring becomes possible. Additionally chemical fingerprinting of unknown substances is practicable. The measurements showed that all thin films on the investigated materials glass, silicon, and AlMg3 were detectable so the CARS method can be used in practice.

Coherent Raman scattering imaging with CARS provides a powerful tool to detect thin silicone films. Potentially there are some limitations. Especially very thin films in the nanometer range are under suboptimal circumstances not detectable because of the low Raman signal in combination with the susceptibility of CARS to non-resonant substrate background, which can mask the Raman signal and complicate quantitative analysis.

Those issues will be investigated in future studies. The quantification of the layer thickness will be examined, beginning with determining the lowest detection limit of CRS with CARS. Therefore, PDMS monolayers will be created with a Langmuir–Blodgett trough and inspected with CRS. Furthermore, the impact of thin silicone films on adhesive bonding will be studied with practical test series involving various surface coverages and different surface treatments to determine the effect of PDMS contamination and the options to handle it.
